# The association between the end of court-ordered school desegregation and preterm births among Black women

**DOI:** 10.1371/journal.pone.0201372

**Published:** 2018-08-22

**Authors:** Menghan Shen

**Affiliations:** Institute of Advanced Study, Waseda University, Shinjuku, Tokyo, Japan; Univesity of Iowa, UNITED STATES

## Abstract

Racial segregation, and in particular school segregation, likely plays an important role in affecting health outcomes. To examine this connection, this paper explores the relationship between the end of court-ordered school desegregation and preterm births among Blacks using birth certificate information between 1992 and 2002 (n = 183,178). The end of court-ordered oversight has important implications for the level of racial segregation in schools: If residential segregation remains high, neighborhood-based student assignment plans would naturally increase school segregation. A rise in school segregation may lead to worse educational, labor, and health outcomes among Blacks. Using multiple difference-in-differences framework that exploits variation in exposure to schools that declared unitary status, it finds that school districts’ release from court oversight is associated with a 0.8 percentage point increase in preterm births among Black mothers. This paper contributes to literature that finds that the end of court-ordered school desegregation in the 1990s have negative implications for Blacks. More research should be conducted to understand the causal relationship between school segregation and infant health.

## Introduction

Preterm births are defined as those that occur at less than 37 weeks of gestational age. In 2002, the preterm birth rate among Black women in the United States was 17.6%. The prevalence of preterm births constitute an alarming public health problem. Infants born preterm are at greater risk for a variety of health problems, such as cerebral palsy, mental retardation, and other major disabilities; they are also more likely to have learning difficulties and suffer from behavioral and psychological problems. Further, preterm birth is associated with lower educational attainment, lower income, and a greater likelihood of receiving Social Security benefits [1–7].

The causes of preterm births are poorly understood [8]. Previous research has focused on individual-level maternal risk factors, including single mother status, low socioeconomic status, cocaine use, late entry into prenatal care, and certain biological markers [9]. Although outcomes for Black women are more likely to be influenced by these factors, they do not fully explain why Black infant have elevated rates of preterm rates [10, 11]. The causes of poor infant health among Blacks are likely to be multifactorial, involving economics, health behaviors, social environments, community dynamics, and segregation [12, 13].

Racial segregation, and in particular school segregation, likely plays an important role in determining infant health outcomes as measured by preterm births and low birth weight. There is a consensus that segregation is one of the leading reasons for elevated rates of poor health outcomes among Blacks [14]. School segregation can affect infant health outcomes directly by affecting the socioeconomic status of families. Extensive research has also documented the causal relationship between school segregation and Blacks’ academic achievement, labor market outcomes, health, and crime [15–19]. Higher levels of parental education, higher family income, and the presence of a father in the household improve infant health outcomes [9, 20]. For example, women with more education have higher earnings, an increase in child quality [21], and an improvement in women’s ability to understand and adopt healthier pregnancy behaviors [20], leading to healthier infants.

In addition, there is a growing body of literature documenting the possible importance of stress as a pathway by which racial segregation affects infant health [22–24]. Segregation is associated with higher levels of racial discrimination, crime, and poverty [15, 17], which may lead to higher stress levels among Black women [25–27]. Exposure to poverty or discrimination-related chronic or acute stressors leads to increased production of corticotropin-releasing hormone, which can lead to preterm births [28–30]. Further, stress hormones can depress immune function, leading to a higher probability of infection [31, 32]. Previous research has demonstrated the effects of neighborhood segregation on infant health outcomes [33–35]. For example, Osypuk and Acevedo-Garcia (2008) found that Black infants in hypersegregated metropolitan areas, defined as those falling above 0.6 on segregation measures, are more likely to be born preterm [34]. School segregation can play a similar role in infant health through its effects on stress.

This paper examines the relationship between the end of court-ordered school desegregation in the 1990s and preterm births. Before 1954, school districts were segregated by law or by residential patterns. The 1954 Brown v. Board of Education case and subsequent decisions over the next two decades eliminated de jure segregation in the South and compelled districts to integrate. However, these decisions were not meant to be permanent (Board of Education v. Dowell, 1991). The Supreme Court issued three decisions in the early 1990s that made the release of schools from court oversight easier [36–38]. An increasing number of school districts started to have their cases dismissed in the early 1990s, meaning that they were free to again use neighborhood-based assignment plans once they reached unitary status.

The end of court-ordered oversight has important implications for the level of racial segregation in schools: If residential segregation remains high, neighborhood-based student assignment plans would naturally increase school segregation. Several empirical studies have validated this concern, finding that school segregation increased in districts that were released from court orders [36–38]. A rise in school segregation may lead to worse educational outcomes for Blacks through two channels: a decrease in the quality of peer effects, as measured by socioeconomic status, and a decrease in education resources, such as less experienced teachers [37]. Lutz (2011) found that the termination of school desegregation plans increases the rate at which Black students drop out of school [37]. In addition, Billings et al. (2014) found that when the end of court-ordered school desegregation increases school segregation, it reduces students’ achievement test scores, reduces their educational attainment, and increases their criminal activity [15]. These prior studies suggest that the end of court-ordered school desegregation in the 1990s could have negative intergenerational implications for Blacks.

Using birth certificate data and a multiple difference-in-differences framework that exploits variation in exposure to schools that declared unitary status provides an excellent opportunity to understand the associations between court-ordered school desegregation and preterm births. This is one of the first empirical papers that examines the link between the end of school desegregation and health outcomes.

## Materials and methods

### Study population

This paper describes a population-based cohort study using data from individual vital statistics natality records covering virtually all live births among Black mothers from 1992 to 2002 in the United States [39]. All data were fully anonymized before the author accessed them. Birth certificates provide information on maternal age, race, maternal education, and county of residence. In addition, they provide information on birthweight, birth order, and gestational age, which is calculated using the self-reported first day of the mother’s last menstrual period.

The study only includes first-time mothers in the analysis because having a child with poor infant health outcomes can affect decisions on whether to have subsequent children, so including later births would complicate the analysis. The study only includes singular births. It does not exclude births with complications. There are 2,194,233 births among Black mothers between the years of 1992 and 2002 that meet these criteria.

In constructing the sample, the author linked information on mothers’ current county of residence with information on the timing of schools’ release from court oversight. Even though the release from court-ordered school desegregation took place at the school district level, the author analyzes the relationship between release from court oversight and infant health outcomes at the county level because doing so mitigates concerns about upward bias driven by women’s possible systemic migration within counties. In creating the sample of schools that were released from court oversight, the author drew on Reardon et al.’s (2012) [38] database of school districts that were ever subject to a court-ordered desegregation plan. The author identified 45 counties with school districts that were released from court oversight between 1992 and 2002 and that have more than 100,000 people in the county (S1 Table). This covers counties in 20 states: Alabama, California, Colorado, Delaware, Florida, Georgia, Illinois, Indiana, Kansas, Kentucky, Maryland, Michigan, Mississippi, New York, North Carolina, Ohio, Oklahoma, South Carolina, Tennessee, and Texas. There are 1,118,523 births among Black mothers that gave births in these 45 counties. This sample constitutes 51% of the births among Black mothers who gave births between the years of 1992 and 2002. The sample selection process is presented in S2 Table.

Further, the author restricted the sample to women who gave births in the same state where they were born. There are 779,163 Black mothers that meet this criteria, which constitutes 70% of first time births among Black mothers who gave births in the 45 counties of interest. For women who were born and gave birth in different state, most of these women may not go to the school in the county where they gave birth. Inclusion of this group of women is likely to bias the coefficients towards zero.

This paper examines the relationship between the end of school desegregation and infant health among women who gave birth between 1992 and 2002. The window of analysis is restricted to include women who attended school within five years (before or after) of when the school district was released from court oversight. In other words, the sample includes the childbearing outcomes from 1992 to 2002 for women who were between 13 and 23 years old when the end of court oversight took place in the school district in their county. This sample, including 181,833 births, constitutes 24 percent of births among first time Black women gave births between 1992 and 2002 in the 45 counties selected and gave births in the same state where they were born in. I also extend the window of analysis to include women who attended schools within 10 years (before and after) of when the school districts were released from court orders. This sample includes 183,178 births and constitutes 38 percent of births among first time Black women who gave births between 1992 and 2002 in the 45 counties selected and gave births in the same state where they were born in.

### Exposure assessment

The exposure variable is defined as ever being exposed to a desegregated school that was released from court oversight. This variable was dichotomized as binary variable: 1 if women were younger than 18 when the school was released from court-ordered desegregation; 0 if women were older than the age of 18 when the school was released from court-ordered desegregation. In other words, I am comparing the childbearing outcomes from 1990 to 2000 for the women who attended schools when the end of court oversight took place in the school district in their county versus women who attended only desegregated schools.

### Statistical analysis

This paper takes advantage of the variation in the timing of school districts’ release from court oversight at the county level. It exploits a similar source of variation as Liebowitz (2018) [40] and Reardon et al. (2012) [38] and uses the same empirical strategy as Guryan (2004), Johnson (2015), Reber (2005), and Shen (2018) [17, 41–43]. The timing of release from court oversight is considered random due to the unequal caseloads across district courts, the varying and somewhat unpredictable duration of the release process, the varying court approaches, and the timing of appeals from interested parties [15]. In addition, since school desegregation took place from the elementary school level to the high school level, it only affects students who were of school age at the time desegregation was implemented.

This paper adopted a multiple difference-in-differences approach that exploits variation in the timing of school districts’ release from court oversight and variation in women’s exposure to desegregated schools. Thus, there are women from the same cohort who are exposed to the treatment (i.e., who attended schools that were released from court-ordered school desegregation) in some counties but not others. There are also some women from a given county who are exposed to the treatment and others from the same county who are not exposed to the treatment. This variation allows for the adoption of cohort fixed effects and county fixed effects.

More specifically, the author built a regression model and included county fixed effects and cohort fixed effects. The main estimating equation on infant health is as follows:

$$y_{ijk}=\alpha_{1}+\beta_{1}Release_{ijk}+\gamma_{k}+\theta_{j}+\epsilon_{ijk}$$ (1)

In this equation, *y*_*ijk*_ refers to a dummy variable that represents whether an infant was preterm. *Release*_*ijk*_ equals 1 if the mother was exposed to schools that were released from court-ordered school desegregation and 0 otherwise. *γ*_*k*_ refers to the year *k* in which the mother was born. *θ*_*j*_ refers to the county *j* where the mother gave birth. It clusters the standard error at the county level.

County fixed effects include a dummy variable for the county where the mother resided when she gave birth; this variable absorbs county characteristics like geographic locations that were constant over time.

Cohort fixed effects include a dummy variable for the year of the mother’s own birth and allow for secular changes over cohorts. For example, if later cohorts were on average more progressive than earlier cohorts, cohort fixed effects would be able to absorb the cohort differences. Cohort fixed effects are also important because counties are unbalanced at the level of infant’s birth year, meaning that since school desegregation takes place in different years in different counties, the actual number of years included in the analysis varies by county. This can be potentially problematic because compared with women in the treatment group, women in the control group are younger when they give birth, since the dataset cannot observe births that took place after 2002. Since cohort fixed effects compare women who were born in the same year, they can absorb the differences in mothers’ age at childbirth resulting from the unbalanced panels of the truncated dataset.

To illustrate the empirical strategy using a simple difference-in-differences approach, if school district being released from court-order took place in 1993 in Lee County, Florida, the treatment group is the cohort that was born in 1976 because women in this group were aged 17 at the time of school being released from court-order. The control group is the cohort that was born in 1974 because women in this group were aged 19 at the time of school being released from court-order. If Dade County, Florida, desegregated in 1991, both the 1976 cohort and the 1974 cohort are in the control group because women in these two groups were already 19 and 21 at the time of school being released from court-order. Thus, to examine the relationship between school being released from court-order and infant health, I can compare the difference in fertility outcomes between the 1976 cohort in Lee and Dade County, which helps control for cohort differences, and difference in fertility outcomes between 1974 cohort in Lee and Dade County, which helps control for county differences. As I have multiple counties and multiple cohorts, I present a multiple difference-in-differences approach across cohorts and counties.

Further, this analysis controls for county-specific year trends, which absorb any county-level changes that are linear over time. If there were non-linear changes that were driving both the timing of the release of school districts from court oversight and infant health outcomes, this study would be subject to omitted variable bias. However, since the timing of court-ordered release is somewhat random, it is possible that there are no endogenous factors that determine both the timing of court-ordered release and infant health. For the main analysis, this paper did not include any information on the mother’s education or age as controls because they are potentially affected by school desegregation. All the analyses in this paper were conducted in Stata (version 14).

## Results

Table 1 provides descriptive statistics for the sample. There are 778,406 births that took place between 1992 and 2002 among first-time Black mothers. There are 183,178 observations for the main analysis that focus on women who were between 13 and 23 years old when the end of court oversight took place in the school district in their county. Sixteen percent of the sample gave birth prematurely, and 12 percent of the births were low weight. Mothers had 11 years of education on average. Sixty-nine percent of first-time mothers were teenagers, and 68% adopted prenatal care in the first three months of pregnancy. Thirty-two percent of births were to teenage fathers.

**Table 1:** Descriptive table.

	1992-2002 Births		Treatment and Control		Treatment		Control	
	Mean	Observation	Mean	Observation	Mean	Observation	Mean	Observation
Preterm Births	15.9	771,933	16.3	181,833	16.9	79,155	16.8	102,678
Low Birthweight	12.2	778,406	12.4	182,945	12.8	79,786	12.2	103,159
Mother’s Education	11.8	766,663	11.1	182,932	10.9	78,381	11.3	101,819
Mother’s Education by Category								
0-8 Yrs	5.4	41,923	8.3	15,228	10.2	8,138	6.9	7,090
9-11 Yrs	33.4	260,107	42.5	77,803	46.7	37,320	39.2	40,483
12 Yrs	33.8	263,668	32.7	59,813	30	23,961	34.7	35,852
13-15 Yrs	18.3	142,736	12.5	22,858	9.8	7,864	14.5	14,994
16 Yrs and Over	7.5	58,229	2.5	4,498	1.4	1,098	3.3	3,400
Not Stated	1.6	12,500	1.6	2,978	1.9	1,499	1.4	1,479
Mother’s Age	20.7	779,163	18.5	183,178	17.9	79,880	19	103,298
Mother’s Age by Category								
Under 19 Yrs	50.7	93,031	69	126,895	76.6	61,256	63.5	65.639
20-24 Yrs	32.7	59,813	26.9	49,328	22	17,513	30.8	31,815
25-More	4.1	7,476	4	6,955	1.4	1,111	5.	5254
Father’s Age	24.7	398,370	22.1	75,365	21.5	30,911	22.6	44,454
Father’s Age by Category								
Under 19 Yrs	12.1	89,663	14	24,191	14.3	11,476	12	12,663
20-24 Yrs	18.44	143,703	18.3	33,567	17.4	13,873	19	19,694
25-More	20.56	165,005	8.7	17,607	7.3	5,562	12	12,045
Unknown	48.9	380,793	59	107,813	61	48,969	57	58,844
Prenatal Care	69	779,163	67.9	183,178	66.3	79,880	69.2	103,298

Preterm births are defined as those occurring at or before 37 weeks of gestational age. Low birthweight is defined as a weight of 2,500 grams or less at birth.

According to the author’s calculation, among all first-time births by Black mothers between 1992 and 2002, the percentage of teenage mothers is 51 percent. In comparison, the percentage of teenage mothers in the sample studied is 69 percent. This is because the sample includes the childbearing outcomes from 1992 to 2002 for women who were between 13 and 23 years old when the end of court oversight took place in the school district in their county. Thus, the sample studies only mothers who are relatively young. Further, as compared to women in the treatment group, women in the control group are younger when they give births since the dataset cannot observe births that took place after 2002. Thus, it is important to adopt cohort fixed effects and compare the births outcomes among women who were born in the same year. Also, it is possible that the effect of the release of school districts from court-ordered desegregation may be different among Black women who give births at an older age. Thus, the interpretation of this analysis is limited to only women studied in this sample.

Table 2 presents results for preterm births using different controls. Model 1 is a univariate analysis that does not control for any confounding variables. Model 2 controls for county fixed effects. Model 3 further controls for cohort fixed effects. Model 4 further controls for county-specific year trends. Models 5 and 6 stratify the population as Southern and non-Southern, respectively.

**Table 2:** The relationship between school districts’ release from court oversight and preterm births (percentage change) among Black women.

	Preterm Births					
	(1)	(2)	(3)	(4)	(5)	(6)
Desegregation end (largest sch dist)	1.182***	1.773***	0.795**	0.895***	0.567	0.845**
SD	(0.175)	(0.189)	(0.346)	(0.347)	(0.816)	(0.410)
Observations	181833	181833	181833	181833	45880	135953
County Fixed Effects		Y	Y	Y	Y	Y
Cohort Fixed Effects			Y	Y	Y	Y
County-Specific Cohort Trends				Y	Y	Y

The outcome of interest is percentage change in preterm rates. Exposure to the end of desegregation equals 1 if the mother was in school when the school district was released from court-ordered desegregation and 0 otherwise. The sample includes first births by Black mothers who were exposed to -5 to 5 years of treatment. Standard errors are clustered at the county level.

*p < .10.

**p < .05.

***p < .01.

Model 1 suggests that school districts’ release from court orders is associated with a 1.2 percentage point increase in preterm births among Black mothers without any controls. Model 2 suggests that school districts’ release from court orders is associated with a 1.8 percentage point increase in preterm births among Black mothers with county fixed effects only. The estimates are both statistically significant at the 1 percent level. Model 3 suggests that school districts’ release from court orders is associated with a.8 percentage point increase in preterm births among Black mothers; this is statistically significant at the 5% level. Model 4 suggests that the results are robust to county-specific year trends: It shows that the release from court orders is associated with an increase in preterm births by 0.9 percentage points; it is statistically significant at the 1 percent level. Models 5 and 6 suggest that while the end of school desegregation is not associated with changes in preterm births in the South, it is associated with 0.8 percentage point increases in preterm births in the non-Southern regions.

Table 3 presents the association between school districts’ release from court oversight and infant health adjusted for years of maternal education, the probability of the child having a teenage mother, the probability of the child having a teenage father, the probability of the mother receiving prenatal care in the first three months of pregnancy, and the probability of nonreporting of the father’s race, in addition to all the controls mentioned above. The estimated coefficients adjusted with the controls range from 0.8 to 0.9 percentage points, and they are statistically significant at the 5 percent level.

**Table 3:** The relationship between school districts’ release from court oversight and preterm births (percentage change) among Black women: Adjusting other factors.

	Preterm Births						
	(1)	(2)	(3)	(4)	(5)	(6)	(7)
Desegregation end (largest Sch Dist)	0.893** (0.411)	0.892** (0.409)	0.839** (0.409)	0.864** (0.409)	0.849** (0.410)	0.807** (0.410)	0.922** (0.411)
Observations	133964	135953	135953	135953	135953	135953	133964
Controls	Maternal Education	Teenage Mother	Teenage Father	Prenatal Care	Married	Father Race Unreported	All
County Fixed Effects	X	X	X	X	X	X	X
Cohort Fixed Effects	X	X	X	X	X	X	X
County-Specific Cohort Trends	X	X	X	X	X	X	X

The outcome of interest is percentage change in preterm rates. Exposure to the end of desegregation equals 1 if the mother was in school when the school district was released from court-ordered desegregation and 0 otherwise. The sample includes first births by Black mothers who were exposed to -5 to 5 years of treatment. Standard errors are clustered at the county level.

*p < .10.

**p < .05.

***p < .01.

S3 Table presents a robustness analysis. Model 1 extends the window of analysis to include women who were 18 years old ten years before and after the year of release from court oversight. The results indicate that release from court oversight is associated with a 0.7 percentage point increase in preterm births. The coefficient is statistically significant at the 5 percent level. Model 2 restricts the window of analysis to include women who were 18 years old three years before and after the year of release from court oversight. The results indicate that release from court oversight is associated with a 1 percentage point increase in preterm births. This coefficient is also statistically significant at the 5 percent level. In Model 3, it defines county-level release from court oversight using the latest date of release by school district. In Model 4, it defines county-level release from court oversight using the earliest date of release by school district. The results are not sensitive to how I specify the sample or the treatment: S2 Table continues to find that the end of school desegregation is associated with increases in preterm births. The estimates range between 0.7 to 0.8 percentage points increase in preterm births.

S3 Table Column 5 examines whether the release of school from court-order is associated with individual moving at all within the last five years. It adopts the same empirical model as Eq (1) and changes the outcome of interest to mobility. It finds that the being exposed to schools released from court orders is not statistically significantly related to mobility.

## Discussion

This paper shows that the end of school desegregation is associated with a 0.8 percentage point increase in preterm births among Black mothers. This effect size is similar to the positive effects of the court-ordered school desegregations that took place from the 1960s to the 1980s [43]. Shen (2018) found that school desegregation reduced preterm births among Black mothers by 0.62 percentage points [43]. However, Shen also found that the benefits of school desegregation were concentrated in the South, while this paper indicates that the loss is concentrated in non-Southern regions. This finding is consistent with previous literature suggesting that negative effects from school districts’ release from court oversight are more pronounced in non-Southern regions. For example, Lutz (2011) found an impact of release from court oversight on Black dropout rates only in non-Southern regions. In addition, Liebowitz (2017) found that the effect of declaration of districts as unitary led to a 2-percentage-point increase in dropouts among Blacks in the South and a 4-percentage-point increase in dropouts among Blacks outside of the South. Thus, it is not surprising that the intergenerational effects of release from court oversight are stronger in non-Southern regions.

There are a number of reasons why the end of school segregation levels is associated with negative outcomes in education, which in turn can have negative long-term implications for Blacks, including implications for their health outcomes. First, the end-of-school desegregation changes the racial composition of the school [17, 41]. Changes in racial composition leads to changes in peer effects: depending on how Blacks perceive Whites, fewer Whites could be good or bad for school quality [44]. Peer effects can play an important role in affecting education, health, and employment outcomes [45]. Second, changes in the level of school segregation may shift the education resources schools receive. School resources may be an important determinant in students’ education and labor market outcomes [46]. Third, it is possible that the end of school desegregation increased the stress experienced by Black women through its effect on the racial discrimination, crime levels, and poverty rates [15, 17].

Table 3 presents descriptive analysis on the possible mechanisms. The results indicate that the relationship between the end of court-ordered school desegregation and preterm births is not attenuated by the inclusion of control variables for years of maternal education, the probability of the child having a teenage mother, the probability of the child having a teenage father, the probability of the mother receiving prenatal care in the first three months of pregnancy, or the probability of nonreporting of the father’s race. This provides suggestive evidence that these factors are not channels for the association between the end of school desegregation and infant health.

This paper has several strengths. First, it utilizes an administrative dataset that covers infant health for virtually for births in the United States between 1992 and 2002. Second, it provides a quasi-experimental setting to explore the relationship between the end of school desegregation on infant health outcomes. However,given the limitation of the data, threats to validity exist in this paper: since the dataset does not provide information on where the mother obtained education, I have to use information on the county of residence when the women was giving birth to assign treatment. I examine the issue of mobility directly using the Census Bureau’s 2000 1-percent Public Use Microdata Sample [47]. There are 314,755 Black individuals in this dataset. Forty-two percent lived in the same home for the last five years, 35 percent moved within the state (including within counties), and 7 percent moved across states. If Black mothers stayed within a county, it would not be a problem for this analysis. In addition, even if Black women moved within states across counties, as long as Black women with higher socioeconomic status did not move systematically in response to school districts being released from court-ordered school desegregation, it would not lead to an upward bias in the estimate of interest. S2 Table, Column 5 shows that schools’ release from court oversight is not associated with individuals moving at all within the last five years. This provides some assurance that the results on infant health are not driven by Blacks’ endogenous migration Second, women in the sample are relatively young when they give birth because the study is limited to birth information from 1992 to 2002 and does not allow for study of their lifetime fertility outcomes. Hence, the study examines the relationship between the release from court-ordered school desegregation and preterm births conditional on Black women giving birth during this period. It is possible that the effects of school districts’ release on infant health are different among Black women who give birth at an older age. It is also possible that school districts’ release affects the timing of childbearing.

This paper contributes to literature on education policies and health outcomes [20, 48–50]. It is also related to literature that suggest differences in infant health outcomes may be rooted in social inequalities. More research should be conducted to understand the causal relationship between school segregation and infant health. Further, future research should differentiate the mechanisms of how school segregation affects infant health before public policy makers can adopt interventions.

## Supporting information

S1 Table List of school districts that are released from court oversight. (PDF)

S2 Table Sample selection (All samples are restricted to 1st time births among Black mothers between 1990 to 2000). (PDF)

S3 Table The relationship between school districts’ release from court oversight and preterm births among Black women: Robustness analysis. (PDF)
