# Supplementary material for: The association between the end of court-ordered school desegregation and preterm births among Black women
Source: PLoS One. 2018 Aug 22;13(8):e0201372. doi: 10.1371/journal.pone.0201372 (PMC6104921; doi:10.1371/journal.pone.0201372)
Supplement: S1 Table — (PDF) [file pone.0201372.s001.pdf]

Table S1 List of school districts that are released from court oversight

State	County	Sch Dist	Total Blacks Students in Sch Dist	Year of Dismissal in Sch Dist
Alabama	Mobile	Mobile County Sch Dist	117861	1997
Alabama	Montgomery	Montgomery County Sch Dist	86732	1993
Alabama	Tuscaloosa	Tuscaloosa City Sch Dist	22565	2000
California	San Diego	San Diego Unified	94681	1998
California	Santa Clara	Palo Alto Unified	6735	1998
Colorado	Denver; coext. with Den	Denver County 1	60066	1995
Delaware	New Castle	Christina School District	20780	1996
Florida	Broward	Broward Csd	193447	1996
Florida	Duval	Duval Csd	162648	2001
Florida	Hillsborough	Hillsborough County School District	109640	2001
Florida	Lee	Lee County (Fort Myers)	22184	1999
Florida	Pinellas	Pinellas County School District	65486	2000
Florida	Polk	Polk County School District	54385	2000
Georgia	Chatham	Chatham County School District	82607	1994
Georgia	De Kalb	Decatur City School District	199089	1996
Georgia	Muscogee	Muscogee	64249	1997
Illinois	Winnebago	Rockford School Dist 205	22084	2001
Indiana	Marion	M S D Decatur Township	134145	1998
Kansas	Shawnee	Topeka Public Schools	11357	1999
Kansas	Wyandotte	Kansas City	42529	1997
Kentucky	Jefferson	Jefferson County	113062	2000
Maryland	Prince George's	Prince Georges County Pub Schs	368420	2002
Michigan	Berrien	Benton Harbor Area	19975	2002
Michigan	Genesee	Flint City School District	67662	2002
Michigan	Oakland	Ferndale Public Schools	31130	2000
Mississippi	Harrison	Biloxi Public School Dist	8646	2002
Missouri	St. Louis city	St. Louis City	188701	1999
New York	Erie	Buffalo	100576	1995
New York	Westchester	New Rochelle	26547	2002
North Carolina	Mecklenburg	Charlotte-Mecklenburg Schools	134465	2002
Ohio	Allen	Lima City Sd	8816	1999
Ohio	Cuyahoga	Cleveland Municipal Sd	234224	1998
Ohio	Hamilton	Cincinnati	142930	1991
Ohio	Lorain	Lorain City School	8708	1994
Ohio	Montgomery	Dayton	78066	2002
Oklahoma	Oklahoma	Oklahoma	56609	1991
South Carolina	Charleston	Charleston County School District	101607	1994
Tennessee	Davidson	Nashville-Davidson County Sd	119273	1998
Tennessee	Shelby	Memphis City School District	339901	1992
Texas	Bell	Temple Isd	7582	2000
Texas	Dallas	Dallas Isd	261986	1994
Texas	Harris	Aldine Isd	50419	2002
Texas	Lubbock	Lubbock Isd	15017	1991
Texas	Nueces	Corpus Christi Isd	9723	1992
Texas	Wichita	Wichita Falls Isd	8836	1999
