# Supplementary material for: The association between the end of court-ordered school desegregation and preterm births among Black women
Source: PLoS One. 2018 Aug 22;13(8):e0201372. doi: 10.1371/journal.pone.0201372 (PMC6104921; doi:10.1371/journal.pone.0201372)
Supplement: S2 Table — (PDF) [file pone.0201372.s002.pdf]

Table S2 Sample selection (All samples are restricted to 1st time births among Black mothers between 1990 to 2000)

Sample Selection	Sample Size	% 1st time births in 45 counties
1st time births	2194233	
in 45 counties	1118523	100%
in 45 counties didn't move	779163	69.7%
in 45 counties exposed to -10 to 10 yrs of treatment	295995	38.0%
in 45 counties exposed to -5 to 5 yrs of treatment	183178	23.5%

Supporting information
