# Supplementary material for: The association between the end of court-ordered school desegregation and preterm births among Black women
Source: PLoS One. 2018 Aug 22;13(8):e0201372. doi: 10.1371/journal.pone.0201372 (PMC6104921; doi:10.1371/journal.pone.0201372)
Supplement: S3 Table — (PDF) [file pone.0201372.s003.pdf]

Table S3 The relationship between school districts' release from court oversight and preterm births among Black women: robustness analysis

	Preterm Births (percentage change)				
	(1)	(2)	(3)	(4)	(5)
Desegregation end (largest sch dist)	0.624***	0.958**			0.034
SD	(0.258)	(0.438)			0.057
Desegregation end (last sch dist)			0.836**		
SD			(0.404)		
Desegregation end (first sch dist)				0.741**	
SD				(0.333)	
Observations	293,889	120489	132856	195131	
County Fixed Effects	X	X	X	X	X
Cohort Fixed Effects	X	X	X	X	X
County-Specific Cohort Trends	X	X	X	X	X
Sample	-10 to 10	-3 to 3	-5 to 5	-5 to 5	-5 to 5

The outcome of interest is percentage change in preterm rates.Exposure to the end of desegregation equals 1 if the mother was in school when the school district was released from court-ordered desegregation and 0 otherwise. The sample includes first births by Black mothers who were exposed to -9 to 9 years of treatment in Model 1. The sample includes first births by Black mothers who were exposed to -3 to 3 years of treatment in Model 2. The sample includes first births by Black mothers who were exposed to -5 to 5 years of treatment in Model 3 and 4. Standard errors are clustered at the county level.*p < .10. **p < .05. ***p < .01.
